# *Salmonella* in Retail Food and Wild Birds in Singapore—Prevalence, Antimicrobial Resistance, and Sequence Types

**DOI:** 10.3390/ijerph16214235

**Published:** 2019-10-31

**Authors:** Kyaw Thu Aung, Hong Jun Chen, Man Ling Chau, Grace Yap, Xiao Fang Lim, Mahathir Humaidi, Cliff Chua, Gladys Yeo, Hooi Ming Yap, Jia Quan Oh, Vijitha Manogaran, Hapuarachchige Chanditha Hapuarachchi, Matthias Maiwald, Nancy Wen Sim Tee, Timothy Barkham, Tse Hsien Koh, Ramona Alikiiteaga Gutiérrez, Jorgen Schlundt, Lee Ching Ng

**Affiliations:** 1 Environmental Health Institute, National Environment Agency, Singapore 138667, Singapore; AUNG_Kyaw_Thu@sfa.gov.sg (K.T.A.); hjchen911@gmail.com (H.J.C.); CHAU_Man_Ling@sfa.gov.sg (M.L.C.); Grace_YAP@nea.gov.sg (G.Y.); xiaofang.lim@duke-nus.edu.sg (X.F.L.); Mahathir_Humaidi@nea.gov.sg (M.H.); Cliff_CHUA@nea.gov.sg (C.C.); Gladys_Yeo@nea.gov.sg (G.Y.); Jennifer_YAP@nea.gov.sg (H.M.Y.); Andrew_OH@sfa.gov.sg (J.Q.O.); Vijitha_MANOGARAN@sfa.gov.sg (V.M.); Chanditha_HAPUARACHCHI@nea.gov.sg (H.C.H.); ramona_a_gutierrez@ncid.sg (R.A.G.); 2 Nanyang Technological University Food Technology Centre (NAFTEC), Singapore 637459, Singapore; jschlundt@ntu.edu.sg; 3 School of Chemical and Biomedical Engineering, Nanyang Technological University, Singapore 637459, Singapore; 4 School of Biological Science, Nanyang Technological University, Singapore 637551, Singapore; 5 National Centre for Food Science, Singapore Food Agency, Singapore 718837, Singapore; 6 Department of Pathology and Laboratory Medicine, KK Women’s and Children’s Hospital, Singapore 229899, Singapore; matthias.maiwald@singhealth.com.sg (M.M.); nancy.tee.w.s@singhealth.com.sg (N.W.S.T.); 7 Department of Microbiology and Immunology, Yong Loo Lin School of Medicine, National University of Singapore, Singapore 117545, Singapore; 8 Duke-NUS Graduate Medical School, National University of Singapore, Singapore 169857, Singapore; 9 Department of Laboratory Medicine, Tan Tock Seng Hospital, Singapore 308433, Singapore; timothy_barkham@ttsh.com.sg; 10 Department of Pathology, Singapore General Hospital, Singapore 169856, Singapore; koh.tse.hsien@singhealth.com.sg; 11 National Centre for Infectious Diseases, Singapore 308442, Singapore

**Keywords:** *Salmonella*, prevalence, molecular epidemiology, antimicrobial resistance, food safety, zoonotic, one health

## Abstract

Non-typhoidal salmonellosis is a leading cause of foodborne zoonosis. To better understand the epidemiology of human salmonellosis, this study aimed to determine the prevalence, antimicrobial resistance and sequence types of *Salmonella* in retail food and wild birds (proximity to humans) in Singapore. We analyzed 21,428 cooked and ready-to-eat food and 1,510 residual faecal samples of wild birds collected during 2010–2015. Thirty-two *Salmonella* isolates from food and wild birds were subjected to disc diffusion and multi-locus sequence typing (MLST). *Salmonella* was isolated from 0.08% (17/21,428) of food and 0.99% (15/1510) of wild birds. None of the isolates from wild birds (*n* = 15) exhibited phenotypic resistance, while the isolates from food (47.1%, 8/17) showed a high prevalence of phenotypic resistance to, at least, one antimicrobial. These findings suggested that the avian *Salmonella* isolates had been subjected to less antimicrobial selection pressure than those from food samples. MLST revealed specific sequence types found in both food and wild birds. The study can guide future studies with whole-genome analysis on a larger number of isolates from various sectors for public health measures.

## 1. Introduction

Salmonellosis, caused by Gram-negative bacteria of the genus *Salmonella*, is a leading type of foodborne infection in humans worldwide [1,2]. Contaminated food is likely the primary cause of salmonellosis. Besides foodborne transmission, the literature has shown several human salmonellosis outbreaks that have been associated with wild birds, suggesting a role for zoonotic transmission [3]. In addition to their ability to cause infection, there has been an increasing number of *Salmonella* strains reported as being resistant to commonly used antimicrobials [4,5]. This may significantly affect human health by limiting the choice of antimicrobials for treating severe salmonellosis cases in humans.

In order to strategize public health measures for controlling indigenous cases of salmonellosis, it is important to gather information on the occurrence and distribution of *Salmonella* serovars in a particular setting. Multi-locus Sequence Typing (MLST) was chosen instead of conventional Kauffman-White serotyping, as it is more discriminating and reproducible [6]. When coupled with antimicrobial susceptibility profiles, a greater discrimination between strains can be obtained, in order to provide useful information for surveillance and epidemiological investigations.

In Singapore, non-typhoidal salmonellosis is a leading cause of foodborne diseases [7]. Over the decade since the disease has been made notifiable in 2008, the incidence of non-typhoidal salmonellosis has increased [8]. However, information on the prevalence and characteristics of *Salmonella* species, in cooked or ready-to-eat food and wild birds in Singapore, is limited. In addition, there is a limited number of reports globally describing the prevalence and characteristics of *Salmonella* in wild birds, especially with respect to those in food chain. Although, wild birds would not directly connect to the food chain, there may be potential links between the *Salmonella* strains from food and wild birds. Such information would provide an insight in better understanding the epidemiology of *Salmonella* in the larger environmental ecosystem in relation to human health.

This study, thus, aimed to identify and report on the prevalence, antimicrobial resistance, and sequence types of *Salmonella* species isolated from cooked and ready-to-eat retail food and wild birds. Such information is imperative in guiding further investigations and public health risk management strategies in Singapore and elsewhere.

## 2. Materials and Methods

Two independent epidemiological studies for food and wild birds were conducted during the same study period (2010–2015) as follow.

Isolation of *Salmonella* in cooked and ready-to-eat food: A cross-sectional study was carried out to determine the prevalence of *Salmonella* in cooked and ready-to-eat food. A total of 21,428 cooked or ready-to-eat food samples were analyzed in this study. The food samples were collected by the Food and Water Sampling Unit (FWSU) of the National Environment Agency (NEA) from 2010 to 2015 (study period). The samples were convenience-randomly collected from different types of retail food premises (hawker centers, restaurants, caterers, food courts), across various regions of Singapore, as part of FWSU’s routine food surveillance program. The categories and types of food sampled are shown in Table 1. For each sample, at least 100 g of cooked and ready-to-eat food were collected in either a sterile bag or in its original packaging. Upon collection, all samples were placed in cooler bags with ice and transported to a commercial laboratory accredited under the Singapore Accreditation Council Singapore Laboratory Accreditation (SAC-Singlas) Scheme. All food samples were tested for the presence (per 25 g of food) of *Salmonella* using methods specified in the U.S. Food and Drug Administration Bacteriological Analytical Manual (FDA-BAM) Chapter 5 [9].

Isolation of *Salmonella* in faecal samples from wild birds: A cross-sectional study was carried out to determine the prevalence of *Salmonella* in wild birds. A total of 1510 residual faecal samples from wild birds were collected for the isolation of *Salmonella*. Bird (injured) carcasses were conveniently received frozen by the Environmental Health Institute of the National Environment Agency, Singapore, from 2010 to 2015 (study period) as part of the zoonotic disease surveillance program. These birds were primarily resident birds collected from urban areas and recreational parks. Approximately 1g of faecal matter, after dissection, was incubated with 9 mL of Universal Pre-enrichment Broth at 35 ± 1 °C for 18–24 h. The enriched samples were streaked onto Xylose Lysine Desoxycholate (XLD) agar (Oxoid, UK) and incubated at 35 ± 1 °C for another 18–24 h. Presumptive *Salmonella* colonies were confirmed biochemically using API 20E (bioMérieux, France) and serological latex agglutination tests (Oxoid, UK).

Biobank of *Salmonella* isolates: *Salmonella* isolates obtained from cooked and ready-to-eat food and wild birds were subsequently subjected to antimicrobial susceptibility testing and multi-locus sequence typing (MLST), as described below. All *Salmonella* isolates were stored in Brain Heart Infusion broth (Acumedia, US) with 15% glycerol at −80 °C and were freshly sub-cultured on Tryptone Soy Agar (Oxoid, UK) before antimicrobial susceptibility testing and multi-locus sequence typing (MLST).

Antimicrobial susceptibility testing of *Salmonella* isolates: Susceptibility tests were performed by using the disk diffusion method according to the Clinical and Laboratory Standards Institute guideline (CLSI, 2013) with 11 antimicrobial agents of eight classes; namely amikacin 30 µg (AK30), amoxycillin-clavulanic acid 20/10 µg (AMC30), ampicillin 10 µg (AMP10), ceftriaxone 30 µg (CRO30), ciprofloxacin 5 µg (CIP5), chloramphenicol 30 µg (C30), gentamicin 10 µg (CN10), nalidixic acid 30 µg (NA30), norfloxacin 10 µg (NOR10), sulphamethoxazole-trimethoprim 23.75/1.25 µg (SXT25) and tetracycline 30 µg (TE30) (Oxoid, Basingstoke, UK) [10]. The antibiotics were selected based on their public health importance. Isolates were classified as sensitive (S), intermediate (I) or resistant (R). Multidrug-resistant (MDR) strains were defined as such by phenotypic resistance to three or more antimicrobial classes.

Multi-locus sequence typing (MLST) of *Salmonella* isolates: *Salmonella* DNA was extracted from isolates by using the QIAamp DNA Mini Kit (Qiagen, Hilden, Germany) according to the manufacturer’s instructions. PCR amplifications were then carried out using primers targeting the gene loci (aroC, dnaN, hemD, hisD, purE, sucA, thrA) described in the MLST database (http://mlst.warwick.ac.uk/mlst/dbs/Senterica/documents/primersEnterica_html) [11]. All PCRs were carried out in a final reaction volume of 50 µl. The reaction mix contained 5X reaction buffer (Thermo Scientific, Vilnius, Lithuania), 1 U of DNA polymerase (Thermo Scientific, Lithuania), 0.2 mM of dNTP Mix (1st BASE, Seri Kembangan, Malaysia), 1 μL (10 μM) of each primer (Integrated DNA Technologies, Singapore) and 5 μL of DNA template. The PCR protocol was as follows: initial denaturation at 98 °C for 30 s, followed by 35 cycles of 98 °C for 10 sec, 55 °C for 30 sec, 72 °C for 30 s, with a final extension at 72 °C for 10 min. The amplified fragments were visualised on 2% agarose gels and subsequently; purified and sequenced using BigDye^®^ Terminator v3.1 Cycle Sequencing Kit (Applied Biosystems, Waltham, MA, USA). Raw sequences were then assembled in Lasergene Software version 8.0 (DNASTAR, Madison, WI, USA) and the consensus sequences were compared with those available in the MLST database (http://enterobase.warwick.ac.uk/species/senterica/allele_st_search) to determine allelic numbers and sequence types. *Salmonella* serovars were predicted based on sequence types of strains available in the MLST database [12].

Statistical analysis: The 95% confidence intervals of proportions were calculated using http://vassarstats.net/prop1.html. Z-scores for two-population proportions were calculated using http://www.socscistatistics.com/tests/Default.aspx.

## 3. Results

### 3.1. Prevalence of Salmonella in Cooked or Ready-to-Eat Food and Wild Birds

From the 21,428 cooked or ready-to-eat food samples tested, 17 (0.08%) were positive for *Salmonella* species (Table 1). Nine of these 17 were poultry/egg dishes (Table 1). Of 1510 wild bird carcasses tested, *Salmonella* species were detected in 15 (0.99%) faecal samples. Those birds belonged to Columbiformes (*n* = 3), Passeriformes (8), Pelecaniformes, and (3) Strigiformes (1). The details of wild birds detected with *Salmonella* are shown in Table 1.

### 3.2. Antimicrobial Resistance in Salmonella Isolated from Food and Wild Birds

Nearly half of *Salmonella* isolates from food (47.1%, 8/17) samples were resistant to at least one of the antimicrobials tested in this study (Table 2). In contrast, none of the *Salmonella* isolates from wild birds (*n* = 15) showed phenotypic resistance to any of the antimicrobials. The proportion of *Salmonella* isolates, resistant to at least one antimicrobial, from food samples (47.1%, Z-score 3.0679, *p* < 0.05) was significantly higher than that of isolates from wild birds (0.0%) (Table 2). One of 17 *Salmonella* isolates from a food sample (5.9%) was resistant to three or more antimicrobial classes and thus was considered a MDR strain (ST3633, *S.* Albany) (Table 3).

All nalidixic acid-resistant isolates found were considered as having reduced susceptibility to ciprofloxacin. In addition, we found *Salmonella* isolates with directly-measured intermediate susceptibility to ciprofloxacin in food (52.9%, 9/17) and wild birds (60.0%, 9/15) samples. On the other hand, none of the isolates were phenotypically resistant to ciprofloxacin or norfloxacin (fluoroquinolones), ceftriaxone (third generation cephalosporin), or amikacin (aminoglycoside) (Table 3). 

### 3.3. Distribution of Sequence Types of Salmonella Isolates in Food and Wild Birds

**Figure 1:**
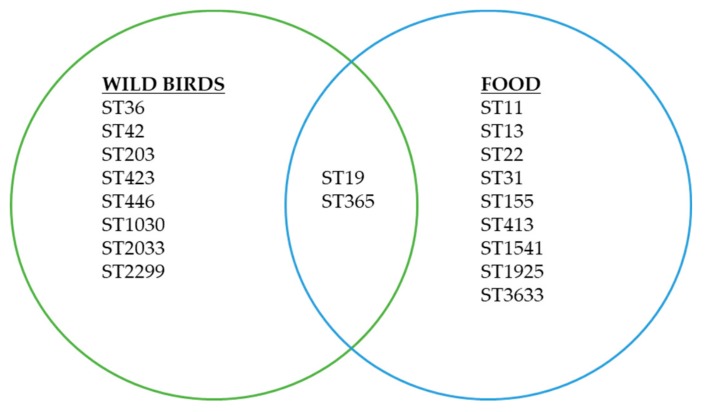
Sequence types and cross sectorial distributions of *Salmonella* isolates in food and wild birds found in this study.

Nineteen different sequence types belonged to 16 predicted serovars were identified in this study. Of 19 sequence types identified, 17 sequence types were found only in either food or wild birds samples (Figure 1). In contrast, 2 sequence types were found in both food and wild birds samples (ST19, ST365) (Table 4).

## 4. Discussion

To the best of our knowledge, this is the first study to estimate the prevalence of *Salmonella* in cooked or ready-to-eat food sold at retail food premises in Singapore. While, a direct comparison of prevalence data between studies was technically challenging, due to the difference in sampling and laboratory methods, the prevalence of *Salmonella* in retail cooked or ready-to-eat food in this study (0.08%) was relatively lower than, or comparable to, that of *Salmonella* reported in overseas countries (Malaysia (17%), Ireland (0.06%–0.1%), Palestine (0.0%), Spain (1.2%–11.1%), Greece (17.9%), Iran (14.0%) and China (1.0%)) [13,14,15,16,17,18,19,20]. Nevertheless, as a large majority (60%) of residents in Singapore dine out at retail food premises at least four times a week, the detection of *Salmonella* in about 1 in 1000 food dishes may constitute a food safety concern [21]. From the *Salmonella*-positive food samples, the majority (9/17) were poultry- or egg-containing cooked dishes. This observation highlights that poultry and eggs, as food ingredients, may be at relatively high risk for *Salmonella* contamination. This suggests that improper cooking and post-cooking contamination are likely contributing factors for the contamination in cooked food.

The majority of wild bird species positive for *Salmonella* in this study were birds well adapted to the urban environments. Wild birds can be natural reservoirs of *Salmonella* [22,23]. Humans can acquire the *Salmonella* infection directly via contact with bird droppings or indirectly via ingestion of contaminated food and food-producing animals [24,25]. Several human salmonellosis outbreaks associated with wild birds have been reported [26,27]. The present study, therefore, reiterates a possible risk of humans acquiring zoonotic salmonellosis through contact with wild birds and their droppings, especially where wild birds are in close proximity to humans.

In addition to their propensity to cause foodborne illnesses, the presence of antimicrobial-resistant *Salmonella* has been increasingly reported around the world [5,28,29]. In this study, nearly half of *Salmonella* isolates from food were resistant to, at least, one of eleven antimicrobials tested. The misuse of antimicrobial agents in food is a key contributing factor for the emergence of resistant pathogens [29,30,31]. Resistant pathogens can further spread to susceptible bacterial populations in the environment through horizontal gene transfer [31]. In contrast, all 15 *Salmonella* isolates from wild birds did not show phenotypic resistance to any of the antibiotics tested. This finding was significant in comparison to the number of resistant isolates from food in this study. The absence of phenotypic antimicrobial resistance in *Salmonella* from wild birds suggests that the birds isolates have not been subjected to antimicrobial selection pressure as much as food isolates [32].

In this study, we detected *Salmonella* isolates from food samples that were resistant to ampicillin, amoxicillin/clavulanic acid, and tetracycline (Table 3). They were susceptible to ceftriaxone, which can be used for the treatment of invasive infections or infections from bacteria resistant to other antimicrobials. It was evident that *Salmonella* isolates from food were resistant to some drugs of choice for the empirical treatment of *Salmonella* or other infections in humans (Table 4). The proportion of food *Salmonella* isolates resistant to these drugs ranged from 5.9% to 35.3%, depending on the type of antimicrobials. The proportion of isolates resistant to nalidixic acid (quinolone, an indicator for reduced susceptibility to fluoroquinolones) was 35.3% (6/17) in food samples. Although, no isolate was resistant to fluroquinolones (ciprofloxacin and norfloxacin), in this study, the finding of *Salmonella* isolates resistant to quinolone (nalidixic acid), as well as isolates with intermediate susceptibility to ciprofloxacin in local food and wild birds samples may be a tell-tale sign for the emergence of fluroquinolones resistance in the local environment (Table 3).

We detected one MDR *Salmonella* isolate (ST3633 *S.* Albany) in a food sample (steamed chicken). MDR *Salmonella* infection is a public health concern, as it can be associated with high morbidity and mortality, which in turn contributes to high healthcare costs and economic burden [33,34]. Although, the prevalence of MDR *Salmonella* strains in this study (5.88%, 1/17 food) was relatively lower than in some overseas studies (32.7%–90.9%), close monitoring is needed for further assessment of the situation. [35,36,37].

Through the application of MLST, we observed that the majority of the sequence types (*n* = 17/19) found in food and wild birds did not overlap. In Singapore, more than 90% of food is imported, and therefore, the sequence types of *Salmonella* found in food, in this study, may represent the introduction of *Salmonella* strains from elsewhere other than from the local environment. Whereas, wild birds most likely acquire *Salmonella* from the local environment through contact with other wild birds’ droppings, or as a result of feeding in contaminated water, or eating *Salmonella*-carrying preys [22].

We detected ST11 (*S.* Entertidis) and ST1925 (*S.* Entertidis) in food samples (ST11 in steamed chicken, sugarcane juice, noodle dark sweet soy-sauce; and ST1925 in chocolate cake, mushroom salad, nasi padang). Both sequence types are known to be geographically widespread and had previously been reported in various sectors, including food, humans, or animals [11]. ST1925 was isolated in an avian sample from a slaughter house in Malaysia in 2012 and from human cases associated with foodborne outbreaks in Singapore (2013–2017) [11,38]. Most of these strains were isolated from ready-to-eat food dishes, which are largely assorted in nature, and thus the ability to track the origins of contamination was limited. Nevertheless, the observations of the *Salmonella* isolates with clinically relevant sequence types (ST1925) in retail food samples suggest that such foods could have been the source of human salmonellosis.

We detected ST42 and ST423 (*S.* Paratyphi B var Java monophasic) isolates in wild birds (black bittern and crow) samples respectively. *S.* Paratyphi B causes paratyphoid fever [39]. Animals, besides human, can be reservoirs of *S.* Paratyphi B [39]. In addition, *S.* Paratyphi B, in particular ST42, has been found in food, feed, fertilizer, and reptiles [40,41]. While, not all variants of *S.* Paratyphi B are capable of causing enteric fever, *S.* Paratyphi B primarily causes gastroenteritis [42], similar to the non-typhoidal strains. The detection of *S.* Paratyphi B in the bird population suggests that wild birds may play a role in the epidemiology of paratyphoidal salmonellosis in Singapore and elsewhere.

Two sequence types, namely ST19 (*S.* Typhimurium) and ST365 (*S.* Weltevreden), were found in both sample types (food and wild birds) suggesting their ability to adapt to, and sustain in, different hosts or types of samples. Besides, the isolates belonged to ST19 and ST365 demonstrated similar antimicrobial susceptibility profiles suggesting the possibility of strain relatedness, common origin, and transmission between food and wild birds. ST19 (*S.* Typhimurium) has previously been found in wild birds and the variant definitive type (DT) 160 was reportedly associated with a prolonged transmission over a 14-year period across different hosts in New Zealand [43,44,45,46,47]. Other variants of *S.* Typhimurium ST19 (DT40 and DT56) isolated from wild birds were genetically similar to isolates from livestock and human cases [26,48]. *S.* Weltevreden is one of the most common serovars reported to be associated with human salmonellosis in tropical countries [7,49,50]. It is largely a monophyletic serovar belonging to the singleton node of sequence type (ST) 365, based on MLST data, and is rarely carrying antimicrobial resistance traits [6,51,52]. *S.* Weltevreden was previously isolated from seafood, shrimps and duck, suggesting an aquatic environment as its potential source of origin [53,54,55,56].

This study analyzed all samples received by the NEA between 2010 and 2015 through the national surveillance program. Although, the sample size of isolates was relatively small. Most *Salmonella* strains in this study were isolated from assorted retail food, and thus, the ability to track the sources of contamination from original ingredients was limited. In addition, the use of MLST provides limited discriminatory power; isolates with identical ST may be distantly related. This warrants future studies by whole-genome analysis of a larger number of isolates from various sources, including human clinical samples.

## 5. Conclusions

This study provides useful information on the characteristics, such as sequence types and antimicrobial resistance profiles, of *Salmonella* in food and wild birds in Singapore. Findings from this study identify specific sequence types of *Salmonella* from food and wild birds that are possibly interrelated, in shaping the epidemiology of salmonellosis, as a basis for further investigations and risk management.

## Figures and Tables

**Table 1:** Prevalence of *Salmonella* in cooked or ready-to-eat food and wild birds.

Types of Samples	Percentage of Samples Positive for *Salmonella* (*N*)	Sample Category (Number of Samples Positive for *Salmonella*)	Sample Information (Number of Samples Positive for *Salmonella*)
Cooked or ready-to-eat food	0.08% (17/21,428) [95% CI: 0.05%–0.13%]	Rice dishes (8)	Duck rice (1), Roasted/steamed chicken (6), Nasi padang (1);
		Noodles (3)	Dry wanton noodle (1), Pasta with white sauce (1), Laksa (1);
		Beverages (2)	Sugarcane juice (2);
		Fastfood (1)	Burger/nugget (1);
		Breads/Confectionery (1)	Chocolate cake (1);
		Snacks (1)	Mushroom salad (1);
		Others (1)	Noodle dark sweet soy-sauce (1)
Wild birds	0.99% (15/1,510) [95% CI: 0.6%–1.63%]	Passeriformes (8)	Black naped oriole (1), Crow (6), Myna (1);
		Columbiformes (3)	Dove (1), Pigeon (1), Rock pigeon (1);
		Pelecaniformes (3)	Bittern (1), Black bittern (1), Black crowned night heron (1);
		Strigiformes (1)	Scops owl (1)

**Table 2:** Percentage of *Salmonella* isolates from food and wild birds resistant to at least one antimicrobial.

Food	Wild Birds
47.1% (8/17) [95% CI, 23.3%–70.8%]	0.0% (0/15)

**Table 3:** Percentage of antimicrobial susceptibility in *Salmonella* isolated from cooked or ready-to-eat food and wild birds.

Antimicrobial Class	Antimicrobial Agents Tested in this Study	Resistance, % (N)		Intermediate-Susceptibility, % (*N*)	
		Food (17)	Wild Birds (15)	Food (17)	Wild Birds (15)
Beta-lactam/beta-lactamase inhibitor combinations	Amoxicillin/clavulanic acid	5.9% (1/17)	0.0%	0.0%	0.0%
Aminoglycosides	Gentamicin	5.9% (1/17)	0.0%	0.0%	6.7% (1/15)
Phenicols	Chloramphenicol	5.9% (1/17)	0.0%	0.0%	0.0%
Folate pathway inhibitors	Trimethoprim/sulphamethoxazole	11.8% (2/17)	0.0%	0.0%	0.0%
Penicillin	Ampicillin	11.8% (2/17)	0.0%	0.0%	0.0%
Tetracyclines	Tetracycline	23.5% (4/17)	0.0%	0.0%	0.0%
Quinolones	Nalidixic acid (Quinolone)	35.3% (6/17)	0.0%	5.9% (1/17)	13.3% (2/15)
Quinolones	Ciprofloxacin (Fluoroquinolone)	0.0%	0.0%	52.9% (9/17)	60% (9/15)
Quinolones	Norfloxacin (Fluoroquinolone)	0.0%	0.0%	0.0%	0.0%
Third generation cephalosporin	Ceftriaxone	0.0%	0.0%	0.0%	0.0%
Aminoglycosides	Amikacin	0.0%	0.0%	0.0%	0.0%

**Table 4:** Characteristics of *Salmonella* isolates from food and wild birds (ST: Sequence type, MLST: Multi locus sequence typing, AK, Amikacin; AMP, Ampicillin; AMC, Amoxycillin-Clavulanic acid; C, Chloramphenicol; CRO, Ceftriaxone; CIP, Ciprofloxacin; CN, Gentamicin; NA, Nalidixic acid; NOR, Norfloxacin; SXT, Trimethoprim-Sulphamethoxazole; TE, Tetracycline). S, Sensitive (Green); I, Intermediate (Yellow); R, Resistant (Red).

Type of Samples	Source	Year of Isolation	ST	Predicted Serovar	AK	AMP	AMC	C	CRO	CIP	CN	NA	NOR	SXT	TE
Food	Steamed chicken	2012	ST11	Enteritidis	S	S	S	S	S	S	S	S	S	S	S
	Sugarcane juice	2010	ST11	Enteritidis	S	S	S	S	S	I	S	S	S	R	R
	Yong Tau Foo dark sauce	2013	ST11	Enteritidis	S	R	S	S	S	I	S	R	S	S	S
	Chocolate cake	2010	ST1925	Enteritidis	S	S	S	S	S	S	S	S	S	S	S
	Mushroom salad	2012	ST1925	Enteritidis	S	S	S	S	S	S	S	S	S	S	R
	Nasi padang	2013	ST1925	Enteritidis	S	S	S	S	S	S	S	S	S	S	S
	Dry wanton noodle	2011	ST22	Braenderup	S	S	S	S	S	I	S	R	S	S	S
	Pasta with white sauce	2014	ST22	Braenderup	S	S	S	S	S	I	S	R	S	S	S
	Roasted chicken	2013	ST13	Agona	S	S	S	S	S	I	S	S	S	S	S
	Steamed chicken	2012	ST3633	Albany	S	R	R	R	S	I	R	R	S	R	R
	Chicken rice	2010	ST1541	Corvallis	S	S	S	S	S	I	S	R	S	S	R
	Duck rice	2014	ST155	London	S	S	S	S	S	I	S	I	S	S	S
	Chicken rice	2014	ST413	Mbandaka	S	S	S	S	S	I	S	R	S	S	S
	Steamed chicken	2012	ST31	Newport	S	S	S	S	S	S	S	S	S	S	S
Wild birds	Black Bittern	2012	ST42	Paratyphi B var Java monophasic	S	S	S	S	S	I	S	S	S	S	S
	Crow	2012	ST423	Paratyphi B var Java monophasic	S	S	S	S	S	I	S	S	S	S	S
	Crow	2013	ST1030	Augustenborg	S	S	S	S	S	S	S	S	S	S	S
	Dove	2015	ST1030	Augustenborg	S	S	S	S	S	I	S	S	S	S	S
	Bittern	2013	ST2299	Stanley	S	S	S	S	S	S	S	S	S	S	S
	Crow	2014	ST2299	Stanley	S	S	S	S	S	I	S	I	S	S	S
	Crow	2012	ST203	Bareilly	S	S	S	S	S	I	S	S	S	S	S
	Black Crowned Night Heron	2014	ST2033	Mgulani	S	S	S	S	S	I	I	I	S	S	S
	Scops Owl	2014	ST446	Hvittingfoss	S	S	S	S	S	S	S	S	S	S	S
	Crow	2013	ST36	Typhimurium	S	S	S	S	S	S	S	S	S	S	S
Food-Wild birds	Pigeon	2013	ST19	Typhimurium	S	S	S	S	S	S	S	S	S	S	S
	Laksa	2015	ST19	Typhimurium	S	S	S	S	S	S	S	S	S	S	S
	Burger/nugget	2010	ST365	Weltevreden	S	S	S	S	S	S	S	S	S	S	S
	Sugarcane juice	2010	ST365	Weltevreden	S	S	S	S	S	S	S	S	S	S	S
	Myna	2011	ST365	Weltevreden	S	S	S	S	S	S	S	S	S	S	S
	Crow	2012	ST365	Weltevreden	S	S	S	S	S	I	S	S	S	S	S
	Rock Pigeon	2014	ST365	Weltevreden	S	S	S	S	S	I	S	S	S	S	S
	Black Naped Oriole	2014	ST365	Weltevreden	S	S	S	S	S	I	S	S	S	S	S
